# Tumor-derived exosomal components: the multifaceted roles and mechanisms in breast cancer metastasis

**DOI:** 10.1038/s41419-021-03825-2

**Published:** 2021-05-26

**Authors:** Yufang Tan, Xiao Luo, Wenchang Lv, Weijie Hu, Chongru Zhao, Mingchen Xiong, Yi Yi, Dawei Wang, Yichen Wang, Haiping Wang, Yiping Wu, Qi Zhang

**Affiliations:** Department of Plastic and Cosmetic Surgery, Tongji Hospital, Tongji Medical College, Huazhong University of Science and Technology, 430030 Wuhan, China

**Keywords:** Breast cancer, Cancer microenvironment, Non-coding RNAs

## Abstract

Breast cancer (BC) is the most frequently invasive malignancy and the leading cause of tumor-related mortality among women worldwide. Cancer metastasis is a complex, multistage process, which eventually causes tumor cells to colonize and grow at the metastatic site. Distant organ metastases are the major obstacles to the management of advanced BC patients. Notably, exosomes are defined as specialized membrane-enclosed extracellular vesicles with specific biomarkers, which are found in a wide variety of body fluids. Recent studies have demonstrated that exosomes are essential mediators in shaping the tumor microenvironment and BC metastasis. The transferred tumor-derived exosomes modify the capability of invasive behavior and organ-specific metastasis in recipient cells. BC exosomal components, mainly including noncoding RNAs (ncRNAs), proteins, lipids, are the most investigated components in BC metastasis. In this review, we have emphasized the multifaceted roles and mechanisms of tumor-derived exosomes in BC metastasis based on these important components. The underlying mechanisms mainly include the invasion behavior change, tumor vascularization, the disruption of the vascular barrier, and the colonization of the targeted organ. Understanding the significance of tumor-derived exosomal components in BC metastasis is critical for yielding novel routes of BC intervention.

## Facts

- Heterotypic exosome transfer in the TME supports the progression, dissemination, and metastasis of breast cancer.
- Exosome-derived components, including proteins, lipids, and nucleic acids, are integral to mediate local and distant cell communication in TME.
- The blocking or inhibition of exosome-derived components might represent a growing body of exciting strategies for combating metastasis.

## Open questions

- How do exosomes derived from tumor act as pivotal carriers involved in tumor invasion and metastasis?
- Can dysregulated exosomal ncRNA and protein levels be used as potential markers of growth and metastasis for breast cancer diagnosis?
- How to develop effective approaches to inhibit tumor metastasis based on exosome components?
- What are the effects and mechanisms of these exosome components on the conventional treatment of breast cancer?

## Introduction

Breast cancer (BC) is the most common female malignancy and one of the leading causes of cancer-related death all over the world^1^. As a highly heterogeneous disease, BC can be classified into various subtypes including luminal A, luminal B, HER2-enriched, basal-like, and normal-like according to the genetic and clinical features^2^. Despite the progress of early detections and relatively effective therapies such as mastectomy, radiotherapy, and chemotherapy, BC is still being confronted with high invasion, metastasis, recurrence rate, and drug resistance^3^. Treatment failure and poor prognosis caused by distant organ metastases are major obstacles to the management of advanced BC patients. Therefore, there is an urgent need to illustrate the key molecular events leading to the malignancy of metastatic breast cancer (mBC)^4^.

**Fig. 1:**
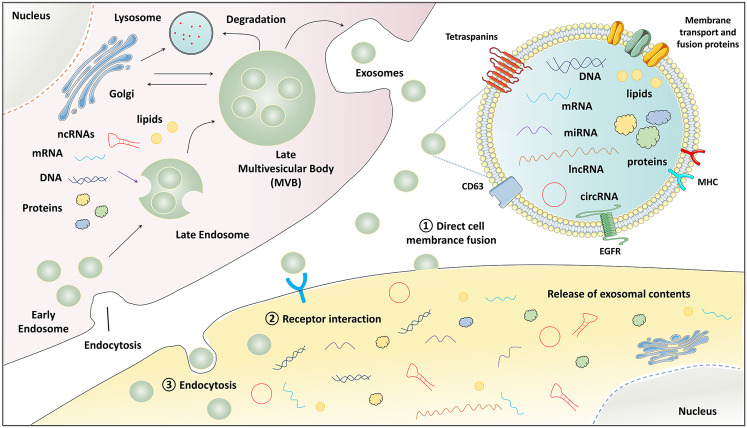
The biogenesis, secretion, and uptake of tumor-derived exosomes. Firstly, the endocytosis results in the formation of early endosomes. Then, the endosomes and selected cargos including nucleic acids, proteins, and lipids are encapsulated into multivesicular bodies (MVBs). Finally, these MVBs fuse with the plasma membrane and release exosomes into extracellular place. The release of exosomal contents (proteins, mRNAs, miRNAs, lncRNAs, circRNAs, and DNAs) to the recipient cells via direct cell membrane fusion, receptor interaction, and endocytosis. noncoding RNA ncRNA, microRNA miRNA, long noncoding RNA lncRNA, circular RNA circRNA, major histocompatibility complex MHC, epidermal growth factor receptor EGFR, late multivesicular body MVB.

Exosomes are defined as specialized membrane-enclosed extracellular vesicles with a particle diameter size of 50–200 nm^5^. In terms of the specific biomarkers, exosomes are enriched in a group of conserved proteins, including the tetraspanin family (CD63, CD9, and CD81), the endosomal trafficking proteins (endosomal sorting complex required for transport (ESCRT)-related proteins/Alix), and heat-shock proteins Hsp60, Hsp70, and Hsp90^6^. The biogenesis and release of exosomes into the extracellular space need to undergo initiation, endocytosis, multivesicular body (MVB) formation, and exosome secretion. Since exosomes are possibly released from almost all cell types, they are widely distributed in the body fluids such as blood, urine, saliva, amniotic fluid, and breast milk, under both healthy and pathological conditions^7^. Importantly, exosomes can deliver multiple functional cargos into the extracellular space, including proteins, lipids, noncoding RNAs (ncRNAs), mRNAs, and DNA fragments^8^. These exosome components act as mediators of cell-to-cell communications locally and systemically by inducing phenotypic alterations in recipient cells (Fig. 1). Therefore, exosomes can impact neighboring or distant cells to modulate antigen presentation, immune function, angiogenesis, cell proliferation, tumor cell migration, and invasion^9^.

**Fig. 2:**
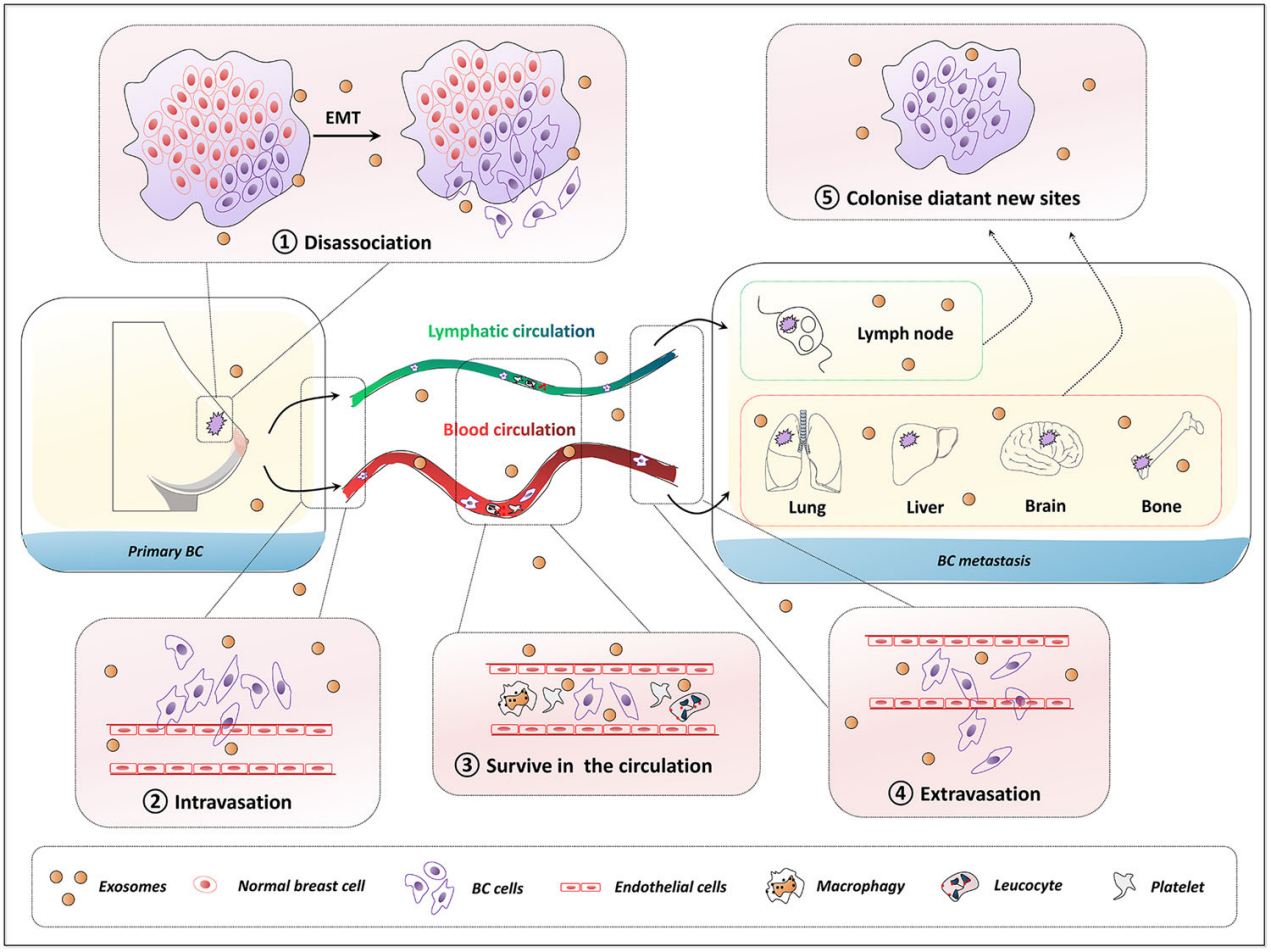
The general multistep process of BC metastasis and exosomes act as regulatory factors. Metastasis is a multistep process by which the primary BC cells undergo epithelial–mesenchymal transition (EMT) and dissociate from the initial site, intravasation, survival in the blood or lymphatic circulation, extravasation, and ultimately colonize distant sites. Exosomes from BC cells are crucial orchestrators to condition the pre-metastatic niche microenvironment in metastatic progression. Breast cancer BC, epithelial–mesenchymal transition EMT.

Metastasis of BC is a multistep evolutionary process by which the cancer cells transcend their programmed behavior to disseminate from the original sites, intravasate into the lymphatic system and blood circulation, and eventually extravasate to colonize distant sites^10^ (Fig. 2). Increasing evidence has shown that exosomes play an irreplaceable role in regulating the BC process and aggressive phenotype^11^. Tumor-derived exosomes have been shown to regulate pre-metastatic niche formation, organotropism, migration, invasion, stemness, and survival. For instance, BC-derived exosomes could promote the ability of BC-cell proliferation, motility, and metastasis, thus generating an enhanced oncogenic phenotype^12^. In addition, Piao et al. reported that BC cell-derived exosomes stimulated macrophage polarization that established favorable conditions for metastatic processes of the lymph node in triple-negative breast cancer (TNBC)^13^. Mechanismly, tumor-derived exosomes are able to condition the pre-metastatic niche microenvironment by manipulating the metastatic cascade through angiogenesis, immune reaction modulation, signal transduction, and genetic intercellular exchange^14^.

The ncRNAs, mainly including microRNA (miRNA), long noncoding RNA (lncRNA), circular RNAs (circRNAs), are defined as genes that are transcribed into RNAs, but not translated into proteins, accompanied with the ability to regulate gene expression at the levels of transcription, RNA processing, and translation^15^. Considering that exosomes are key carriers of juxtacrine and paracrine signaling for mediating metastatic progression, the large number and the versatility of ncRNAs and proteins in exosomes have fueled many studies on their roles in BC metastasis. Herein, we have summarized the multifaceted roles and mechanisms of tumor-derived exosomes in BC metastasis based on these important components. It will help decipher how the tumor-derived exosomes reshape the tumor microenvironment (TME) and the BC metastasis and will provide novel and effective therapeutic strategies for combating mBC.

## Factors promoting tumor-derived exosome biogenesis, release, and uptake for BC metastasis

Cancer metastasis is a complex, multistage process, which eventually causes tumor cells to colonize and grow at the metastatic site^16^. Exosomes derived from tumors and stromal cells have been involved in all stages of cancer metastasis. In addition to the different types of substances loaded in exosomes, the content of tumor-derived exosomes also impacts BC metastasis. Understanding the cellular basis of exosome biogenesis, release and uptake is critically important for developing targeted therapies by blocking exosomes.

The biogenesis of exosomes is a complex multistep process. Firstly, the endocytosis in lipid raft domains of the plasma membrane results in the intracellular formation of early endosomes. Then, under the participation of the Golgi apparatus, the interluminal vesicles (ILV) buds from endosomal compartments and further connect together, forming the MVB, which means that early endosomes transform into late endosomes accordingly. This conversion is accompanied by a cargo sorting procedure for the recruitment of molecular contents into ILVs, including proteins, nuclear acids, and metabolites. It is generally acknowledged that the mechanism of the exosomal formation processes mainly includes two ways: the ESCRT pathway and the ESCRT-independent pathway. The ESCRT pathway is a complex of four proteins (ESCRT-0-III) including Hrs, CHMP4, TSG101, STAM1, VPS4 that promote MVB formation, budding, and cargo distribution^17^. Lipid raft microdomains play a crucial role in the MVB biogenesis in an ESCRT-independent manner^18^. Meanwhile, a family of 4-transmembrane proteins (tetraspanins), such as CD9, CD63, CD81, CD82, and CD97, are also linked to the formation of ESCRT-independent exosomes^19^. These are all key factors in exosome genesis and regulation, indicating the complexity between cell function and exosome activity/quantity.

Finally, some MVBs bind to lysosomes for degradation, while the other MVBs are guided to the cell membrane, anchor, and fuse with the cell membrane, thus releasing internal ILVs to the outside of the cell to form exosomes. The exocytosis of the ILV-containing MVBs is mainly driven by Rab GTPases at the plasma membrane, specifically RAB27A and RAB27B. Ghoroghi et al. identified RalA/B GTPases were a novel molecular mechanism that could control exosome secretion levels via the homeostasis regulation of MVBs^20^. The RalA/B-phospholipase D1 (PLD1)-phosphatidic acid (PA) axis governed exosome biogenesis to a certain extent. RalA and RalB reduced the EV levels of the adhesion molecule MCAM/CD146, which was in favor to disseminate and induce the formation of pre-metastatic niches in a CD146-dependent manner. Additionally, SNARE protein complex, syntaxin 1A, synaptotagmin protein family, Wnt, and Ca2+ also participate in regulating the process of release of exosomes^19^. Interestingly, Pan et al. found alter levels of Ca2+ affected exosome release from MDA-MB-231 cells, and therefore exerted an impact on angiogenesis of human umbilical vein endothelial cells (HUVECs)^21^. Hypoxic tumors exhibit more aggressive phenotypes and are associated with poor patient outcomes in a wide variety of cancers^22^. Hypoxia is an important feature of solid tumors that promotes tumor progression, angiogenesis, and metastasis, potentially through exosome-mediated signaling^23^. King et al. proved that hypoxia promoted the release of exosomes by BC cells to promote survival and invasion, and that this hypoxic response might be mediated by ﻿hypoxia-inducible factor-1α (HIF-1α)^24^. Furthermore, the hypoxically regulated miR-210 was identified to be present at elevated levels in hypoxic exosome fractions. The study of Wang et al. suggested that the HIF-dependent RAB22A gene expression level was upregulated by hypoxia in advanced BC, which promoted microvesicle generation and stimulated BC invasion^25^. In view of the emerging role of tumor cell-derived exosomes in tumor progression, it is of great significance for understanding the inner link of hypoxic and exosome biogenesis.

Asparaginyl β-hydroxylase (ASPH) gene is upregulated and is required for the generation and maintenance of malignant phenotypes in invasive BC^26^. Lin et al. showed ASPH-Notch axis resulted in ﻿matrix metalloproteinases (MMPs)/ADAMs mediated extracellular matrix (ECM) degradation/remodeling, benefiting invasiveness by particularly guiding exosomal delivery of pro-metastatic secretome^27^. The crucial role of ASPH in promoting invasion, aggressiveness, and multi-organ dissemination, making it a potential therapeutic target for BC metastasis. Autophagy and autophagy-related genes (Atg) also occupy prominent roles in oncogenesis, growth, and metastasis of BC^28^. The Atg5 disassociated the V_1_V_0_-ATPase to promote exosome production and tumor metastasis in vivo independent of canonical macroautophagy^29^. Endothelin receptor A (ETA), belonging to a member of the ﻿G protein-coupled receptor family, is related to various effects of BC growth, metastasis, and angiogenesis. ETA was important to interference with BC cells to promote exosome secretion in BC cells. The inhibition of ETA function by antibiotic sulfisoxazole could result in an anti-metastasis effect in mouse models of BC xenografts via suppressing the biogenesis and secretion of exosomes in an ETA dependent way^30^. Whole-genome sequencing data from human tumors found that Munc13-4, a Rab-binding protein, was elevated in BC. Munc13-4 and Ca2+ uptake increased in BC cells after epithelial–mesenchymal transition (EMT), and further promoted exosome release from highly invasive BC cells, inducing metastasis ultimately^31^. It was worth noting that Munc13-4 regulated MVB maturation and generated MVBs competent for exosome release by a Rab11-dependent trafficking pathway. MAP17 was a commonly upregulated protein in inflammation and carcinomas. Furthermore, MAP17 increased the secretion of BC cell-derived exosomes, and also could be released as exosomal cargoes^32^. Thus, this horizontal propagation also increased the EMT in the recipient cells. MAP17 could be considered as a novel target for mBC attributing to its ability to shape tumor evolution.

After release, exosomes can orchestrate on their parental cells, adjacent and distant cells in an autocrine, a juxtacrine and a paracrine manner. When they reached their target cells, the first way of uptake is to recognize receptors on the surface of the cell membrane and activate the corresponding signaling pathways. The second crucial uptake mechanisms of exosomes is endocytosis, such as pinocytosis, phagocytosis and micropinocytosis. Besides, the special lipid raft structure caveolae-mediated endocytosis is also involved in the uptakes of exosome. The third way is to fusion directly with the plasma membrane of the target cells, thus releasing their cargo. It is worth mentioning that uptake quantity of exosome is also important for exosome-mediated tumor progression. For example, Esposito et al. screened the novel RNA aptamer ex-50.T, which specifically recognized exosomes derived from BC cells and inhibited their cellular uptake as well as interrupted exosome-mediated cell invasion, putting forward the possibility of tumor behavior inhibition by reducing exosomal exchange^33^. Besides, several commonly used agents in clinic are postulated to block exosome uptake through various cellular mechanisms, including anticoagulant heparin, antimalarial drug chloroquine, the diuretic amiloride, and antipsychotic drug chlorpromazine^34^.

In addition to the above extrinsic biological factors, it is important that exosome biogenesis and uptake are also influenced by several external factors, such as cell type, cell state, hypoxia, serum conditions, the added cytokines and growth factors and even the separation and purification method of exosomes^35^. Targeting the biogenesis, release and uptake of exosomes might be a potential therapeutic approach to change the course of cancer.

## Tumor-derived exosomal components in BC metastasis

Tumor-derived exosomes deliver into the bloodstream and mediate nonrandom patterns of metastasis^36^. BC metastasis usually develops in multiple organs including lymph nodes, bone, lung, brain, and liver. The selective preference of tumor metastasis to certain tissues is complicated and dependent on several factors including vascular patterns, adhesion factors, the interaction of tumor cells with the matrix at the site of metastasis. It is recognized that organ-specific metastatic potential requires close interaction between cancer cells and TME. Tumor-derived exosomes have been reported to deliver ncRNAs, proteins, and lipids to incorporate into surrounding cells or cells in distant metastatic niches by conditioning pre-metastatic TME.

### Exosomal noncoding RNA

#### Exosomal miRNA

The miRNAs are small ncRNAs with 19–25 nucleotides length and can regulate gene expression posttranscriptionally by binding to the complementary sites in the 3′ untranslated region (UTR) of targeted mRNAs^37^. Tumor-derived exosomes, especially carrying miRNAs, can be diffused to neighboring cells or systemically transported to the distant organ to induce malignant transformation and field cancerization^38^. Interestingly, in addition to carrying miRNA, serum exosomes from BC patients contained dicer and could process pre-miRNAs to generate mature miRNAs^39^. BC exosomes induced transcriptome alterations in recipient cells and tumor formation in a dicer-dependent manner^39^. Totally, exosomes play pleiotropic roles in cancer progression and metastasis, including invasion, angiogenesis, and immune modulation.

##### Exosomal miRNAs regulate the malignant phenotype of BC cells

At early-stage mBC, tumor cells disseminate through both lymphatic and hematogenous systems. Some miRNAs act as tumor suppressors that migrate through the vasculature by the exosome. Kong et al. found that miR-130a-3p was aberrantly downregulated in human BC tissues and exosomes from circulating blood, while the lower levels of exosome-derived miR-130a-3p were related to lymph node metastasis and advanced TNM stage^40^. In vitro, exosomal miR-130a-3p inhibited the cell proliferation, migration, and invasion of human breast cancer stem cells (BCSCs) by directly regulating RAB5B/﻿epidermal growth factor receptor signaling pathways. Li et al. pointed out that miR-770 encapsulated in tumor exosome could be transported into tumor-associated macrophages, and thereby increased the miR-770 expression in macrophages^41^. Additionally, miR-770 overexpression inhibited the migration and invasion of TNBC via targeting of STMN1. Let-7a and c-Myc were negatively correlated with BC. The Let-7a derived from MDA-MB-231 cell exosomes could inhibit the proliferation, migration, and invasion both in vitro and in vivo by silencing the c-Myc expression^42^. It was also found that miR-188-5p inhibited BC-cell proliferation and migration, which was mediated by the downstream target interleukin 6 signal transducer (IL6ST)^43^. This result revealed that miR-188-5p was selectively sorted into exosomes from malignant BC cells, but not into patient serum exosomes. The transfer of miR-155 from BC exosomes could reprogram the metabolism in adipocytes and muscle cells to induce lipolysis and muscle loss, thus leading to cancer-associated cachexia to promote metastasis in BC^44^.

The exosomal miRNA secreted by BC cells can also dynamically promote the malignant phenotype of tumors, which is related to the refractory and progression of tumors. Ding et al. showed that exosomal miR-222 was highly upregulated and correlated to BC patients with lymphatic metastasis^45^. BC exosome-transferred miR-222 contributed to tumorigenicity and metastatic progression of BC cells both in vitro and in vivo, potentially mediated by tumor suppressor gene PDZ and LIM domain-containing protein 2 (PDLIM2) downregulation and consequent NF-kB activation. Yang et al. showed that miR-146a derived from BC exosomes enhanced the transformation of normal fibroblasts (NFs) into cancer-associated fibroblasts (CAFs) via miR-146a/thioredoxin-interacting protein (TXNIP) axis to promote the Wnt pathway in TME, contributing to the accelerated invasion and metastasis of BC cells^46^. Wang et al. confirmed the exosomal miR-1910-3p enhanced the tumorigenicity in the tumor xenograft model^47^. The miR-1910-3p was enriched in BC-cell exosomes and could transfer to mammary epithelial cells and BC cells, ulteriorly to promote growth, metastasis, and autophagy of BC cells by regulating myotubularin-related protein 3 (MTMR3) and activating the NF-κB signaling pathway. The expression levels of miR-200c and miR-141 were higher in plasma from patients with mBC than localized BC or healthy controls, possessing the potential to be biomarkers for early detection of tumor metastasis^48^. These circulating miR-200c and miR-141 probably were secreted or released from circulating tumor cells mediated by exosomes. Moreover, miR-200c and miR-141 were re-regulated by a FOXP3-KAT2B axis in BC cells.

MiR-155 is a well-known oncogenic miRNA, which is often upregulated in BC, and is widely involved in the recurrence, metastasis, and treatment resistance of BC. miR-155 was enriched and could be separated from cancer stem cells (CSCs) and resistant cells, accompanied by the transfer of miR-155 exosomes to BC cells^49^. Exosomes remodeled the migration capacity with enhanced EMT to sensitive cells partly by exosomal transfer of miR-155. The research of Gorczynski et al. confirmed that both miR-155 and miR-205 could potentiate the inflammatory responses to modulate the metastatic growth of BC cells in the lung and liver metastasis model. Increased BC exosomal miR-205 was reported to attenuate BC metastasis while miR-155 had the opposite effect^50^. By bioinformatic approach, Kia et al. found that miR-9 and miR-155 were among the overexpressed miRNAs in highly-metastatic TNBC cells and their exosomes, which was further validated by qRT-PCR^51^. Luciferase assay verified that the BC exosomal miR-9 and miR-155 targeted the UTRs of tumor suppressors phosphatase and tensin homolog (PTEN) and dual-specificity phosphatases 14 (DUSP14), respectively. Interestingly, in their following study, the metastatic behavior of low-metastatic recipient MCF-7 cells with the treatment of highly-metastatic MDA-MB-231 cells exosomes acquired enhanced metastatic phenotype^52^. The miR-155 shuttled by exosomes introduced a novel behavior with the increased ability of cancer development and metastasis. The elevated miR-7641 in BC-derived exosomes was found in the plasma of BC patients with distant metastasis and could promote tumor growth both in vitro and in vivo^53^. MiR-7641 could promote BC-cell proliferation and invasion, and induce epigenetic modulation in recipient cells via exosome transfer.

##### Exosomal miRNAs induce angiogenesis of tumor

Pathological angiogenesis could provide nutrients and oxygen for tumors, thus facilitating the growth and dissemination of cancers and distant metastasis. Exosomes are crucial mediators in their regulation of angiogenesis and cell migration in tumor growth and spread. MiRNAs are emerging regulatory RNAs that can be selectively encapsulated into exosomes and function as a messenger in intercellular communication to regulate tumor metastasis and angiogenesis, and remodel the TME. Feng et al. showed that BC cells secreted exosomal miR-22-3p mediated tumor vessel abnormalization by suppressing transgelin, thus promoting tumor budding and BC progression in vivo^54^. Pan et al. found exosomal miR-145 in MDA-MB-231 cells targeted IRS1 and inhibited the angiogenesis of HUVECs via regulating IRS1/PI3K/Akt/mTOR and IRS1/Raf/ERK pathways^21^. Furthermore, stromal interaction molecule 1 (STIM1), which was a transmembrane protein located in the endoplasmic reticulum, reduced BC exosomal miR-145 to promote angiogenesis and migration.

##### Exosomal miRNAs mediate disruption of vascular barrier

In the process of metastatic and diffusion, BC gains the ability to transmigrate through blood vessels by inducing alterations in the endothelial barrier. As reported by Modica et al. the exosomal miR-939 in TNBC cells increased tumor cell trans-endothelial migration and directly targeted vascular endothelial cadherin (VE-cadherin) in endothelial cells^55^. This work suggested that BC-secreted exosomal miR-939 was involved in the extracellular pro-tumorigenic characteristic and was associated with a worse prognosis in TNBCs. miR-105 was characteristically secreted by mBC cells and was a potent migration regulator by targeting the tight junction protein zona occluden-1 (ZO-1)^56^. In endothelial monolayers, the BC-secreted exosomal miR-105 effectually damaged the integrity of natural barriers and benefited for metastasis. Clinically, miR-105 could be detected in the circulation at the pre-metastatic stage, and its levels in the blood and tumor were correlated with ZO-1 expression and metastatic progression in early-stage BC. Rodríguez-Martínez et al. Before neoadjuvant therapy, exosomal miRNA-21, and 105 expression levels were higher in metastatic versus nonmetastatic patients and healthy donors^57^.

##### Exosomal miRNAs mediate colonization of targeted organ

In the specific organ metastasis, tumor-derived exosomal miRNAs not only show aberrant expression in content, but also exhibit the ability to prepare the pre-metastatic niche with specific organ-oriented invasion ability. miR-20a-5p was overexpressed in BC tissues and the exosomes of MDA-MB-231 cells^58^. This exosomal miR-20a-5p promoted the migration and invasion in MDA-MB-231 cells, and could be transferred to bone marrow macrophages (BMMs) and facilitated the osteoclastogenesis via targeting SRC kinase signaling inhibitor 1 (SRCIN1). BC cell-secreted exosomes might be a prerequisite for pre-metastatic niche generation by promoting osteoclast differentiation and enhancing bone metastasis. Yuan et al. provided evidence that BC patients with bone metastasis showed higher expression of exosomal miR-21 in serum than those without metastasis or with non-bone tissue metastasis^59^. In experimental exploration, exosomal miR-21 derived from high-metastatic SCP28 cells boosted osteoclastogenesis via regulating programmed cell death 4 (PDCD4) protein levels, resulting in the formation of the pre-metastatic niche.

In the lung metastatic mouse model, the BC exosomal miR-183-5p could transfer to macrophages to promote the secretion of pro-inflammatory cytokines interleukin-1b (IL-1β), IL-6, and tumor necrosis factor-α, via repressing phosphatase 2 catalytic subunit alpha (PPP2CA)^60^. Obviously, the knockdown of miR-183-5p in 4T1 exosomes inhibited BC growth and metastasis in the BC lung metastasis model. In another search, mimic miR-33 delivered by 4T1 cell exosome was able to induce M1 polarization in macrophages, resulted in improved antitumor effect^61^. It was known that tissue inhibitors of metalloproteinase 2 (TIMP2) belonged to the TIMP family and could effectively inhibit the invasion and metastasis of tumors. Wang et al. confirmed that in the BC metastatic mouse model, the overexpression of tumor-derived miR-4443 was of high capacity to induce BC liver metastasis^38^. The conveying exosomal miR-4443 could break the natural barriers, which was accompanied by impaired TIMP2 and consequently upregulated MMP-2 in both the primary tumor and metastasis liver sites, resulted in engrafting in the new microenvironment.

The metabolic profile of most cancer cells resulted in increased aerobic glycolysis with lowered mitochondrial oxidative phosphorylation in an aerobic environment, which is known as the “Warburg effect” to favor the uptake and incorporation of nutrients. By secreting vesicles including exosomes with high levels of the miR-122, BC cells suppressed the glucose uptake of non-tumor cells in the pre-metastatic niche, by downregulating the glycolytic enzyme pyruvate kinase^62^. Intriguingly, the in vivo inhibition of miR-122 significantly restored the efficacy of glucose uptake in distant organs, including the brain and lung, and thus reduced the metastasis incidence. That meant, BC-secreted miR-122 reprogrammed glucose consumption in niche tissues and promoted metastasis. By profiling lncRNAs, Xing et al. found that X-inactive-specific transcript (XIST) was significantly downregulated in tumors with breast cancer brain metastasis (BCBM)^63^. Loss of XIST in BC activated MSN-c-Met and reprogrammed microglia via exosomal miRNA-503, which triggered M1–M2 polarization of microglia and suppressed T-cell proliferation to promote brain metastasis. Sharma et al. utilized a novel therapy for BCBM by delivering athermal radiofrequency electromagnetic fields that are amplitude-modulated at breast cancer-specific frequencies (BCF)^64^. It was interesting that BCF treatment could inhibit the growth of brain metastasis in a mouse model. The result showed that BCF suppressed angiogenesis in the TME by inhibiting β-catenin and decreasing the exosomal secretion of miR-1246. Coincidently, the research of Li et al. also confirmed that exosomal miR-1246 was highly expressed in mBC MDA-MB-231 cells compared to nonmetastatic or nonmalignant BC cells^65^. Besides, exosomal miR-1246 boosted cell proliferation and invasion by inhibiting the expression level of cyclin-G2.

Based on the above studies, it is not hard to see that tumor-derived exosomal miRNAs transfer functions or characteristics into recipient cells. These abundant miRNAs are the key mediators for the bidirectional communication of tumor cells and other cell types, involving all the metastasis processes including the tumor cell dissemination from the primary tumor site, TME formation, and tumor cell colonization in a distant organ. These miRNAs are potential candidates for constituting unique molecular features, identifying clinically primary subtypes, and being therapeutic targets of BC.

#### Exosomal lncRNA

LncRNAs are a category of cellular ncRNAs with a length of more than 200 nucleotides. Recently, lncRNAs have become critical components in cancer evolution by playing a driving role in suppressing and carcinogenic functions in prevalent cancer types, such as BC^66^. For example, a lncRNA associated with BCBM (Lnc-BM) is a prognostic indicator of brain metastasis progression in patients with BCBM^67^. Lnc-BM increased Janus kinase-2 (JAK2) kinase activity to trigger STAT 3 phosphorylation, followed by macrophage recruitment and activation, thus promoting BCBMs by mediating communication between BC cells and the brain microenvironment. BC cell-derived exosomal small nucleolar RNA host gene 16 (SNHG16) promoted the activation of the TGF-β1/SMAD5 pathway by sponging miR-16-5p and resulted in the conversion of γδ1 T cells into the CD73+ immunosuppressive subtype^68^. The lncRNA shuttled by exosome modifies the malignant behavior of the tumor showing the potential to be promising biomarkers for the diagnosis and prognosis of mBC.

By using high-throughput sequencing analysis, Feng et al. manifested that a lot of functional involvement of abnormally expressed lncRNAs derived from BC cells induced proliferation and migration of lung fibroblasts, which provided an appropriate environment for the formation of pre-metastatic niche and facilitated tumor pulmonary metastasis^69^. Among them, there were a total of 64 lncRNAs co-increased expressions and 8 lncRNAs co-decreased. Liang et al. identified that hypoxia-responsive lncRNA BCRT1 was upregulated in BC tissues, and the higher expression of lncRNA BCRT1 was associated with aggressive tumor metastasis and poor prognosis^70^. The transmission of lncRNA BCRT1 from BC-derived exosomes could promote M2 polarization and thus enhance M2 tumor-promoting function. LncRNA BCRT1 acted as a sponge for miR-1303 to weaken its silence effect on polypyrimidine tract binding protein 3 (PTBP3) expression. Lu et al. found that lncRNAGS1-600G8.5 was overexpressed in exosomes derived from BC cells which easily metastasized to the brain^71^. More importantly, by transferring lncRNA GS1- 600G8.5, the exosomes from the high brain mBC cells could be internalized by brain microvascular endothelial cells, which disrupted the permeability of the blood–brain barrier (BBB) and promoted the passage of cancer cells across the BBB in vivo. The in vitro assay further confirmed that the exosomal GS1-600G8.5 could destroy the BBB by inhibiting tight junction proteins.

#### Exosomal circRNA

circRNAs are one of the newly discovered ncRNAs, which are connected end-to-end to form covalently closed single-chain circular molecule. As highly conserved RNAs, many circRNAs contain abundant miRNA-binding sites, suggesting that they may function as miRNA sponges. Exosomal circRNAs derived from BC cells mostly act as competing endogenous RNAs (ceRNAs) in recipient cells, as they modulate miRNA-target gene expression and contribute to oncogenesis and development.

Through microarray and RNA sequence techniques, Wang et al. verified a total 1061 of upregulated circRNA and 86 of downregulated circRNA in the exosomes of patients with mBC^72^. Additionally, 5 circRNAs were confirmed to be elevated in mBC patient-derived exosomes by RT-qPCR, and possible circRNA/miRNA interactions and conceivable functional meshwork were proposed using bioinformatics methods. CircRNAs might have crosstalk with more than one miRNA and be associated with several pathways involving in BC migration and invasion. Similarly, Yang et al. verified two significantly differentially expressed circRNAs in exosomes from BC tissues in comparison with non-tumor tissues, where hsa-circRNA-00005795 was downregulated and hsa-circRNA-0088088 was upregulated^73^. Of note, regarding the role of circRNAs as miRNA sponges, a multifunctional network was structured between hsa-circRNA-00005795 and eight miRNAs and between hsa-circRNA-0088088 and 11 miRNAs respectively using three databases. These circRNAs-associated miRNAs were related to signaling pathways in the BC progression, including but not limited to Wnt, estrogen, and TGF-β pathways. Ding et al. verified that circ_0004771 was higher in serum exosomes from BC patients in comparison to healthy patients, and could harbor miR-1253 in BC to upregulate dimethylarginine dimethylaminohydrolase 1 (DDAH1), contributing to the alteration of BC malignant phenotypes^74^. The identity, dysregulation, and function of exosomal circRNAs in BC metastasis are only beginning to be understood, thus further mechanisms are needed to ascertain the intricate roles of circRNAs in mBC.

### Exosomal protein

The proteome profile of exosomes isolated from BC cells contributes to different metastatic potential. Exosomes secreted by mBC cells are enriched in many membrane proteins. As reported by Gangoda et al. that, by proteomic profiling of highly-metastatic 4T1 and nonmetastatic 67NR exosomes, the former exosomes were enriched with membrane proteins including Cd38, Slco2a1, Acsl4, Mtdh, Fgfr, and Tgfbr^75^. It could be inferred that the exosomes contained various protein cargo depending on the host cells metastatic properties and facilitated in the dissemination of the primary tumors to distant sites. The functions of exosomal proteins of BC cells depend on the following mechanisms, including promoting the invasion behavior, responding to the hypoxic microenvironment, inducing tumor vascularization, disrupting vascular barrier, and mediating targeted organ colonization.

#### Exosomal proteins promote the invasion behavior of BC cells

Fibronectin (FN) is considered to be a key player to promote the BC-cell behaviors including cell adhesion, metastasis, and carcinogenic transformation such as EMT^76^. BC-derived exosomal FN was able to facilitate BC metastasis in vivo, with activated focal adhesion kinase/Src-dependent and enhanced production of pro-inflammatory cytokines and metalloproteinase 9 (MMP9)^77^. Furthermore, the neutralization or small interfering RNA silence of tumor-exosomal FN partially reversed the tumor exosome-mediated tumor cell invasion. Didiasova et al. found that the invasion ability of MDA-MB-231 cells was enhanced when co-cultured with the exosome secreted by cells overexpressing enolase-1 (ENO-1)^78^. In addition to the expression level of cell surface-bound ENO-1, the amount of exosomal ENO-1 into the extracellular space was also correlated with the invasive metastatic potential of cancer cells. It indicated that exosomal ENO-1 might gather proteolytic activity on the BC-cell surface or enlarge the cytoplasmic pool of ENO-1, thereby modulating the expression of the genes related to cell proliferation, migration, and inflammation to promote tumor progression. RAB22A was a small GTPase, belonging to the RAB protein family involved in early endosomes, Golgi bodies, and late endosomes. Sun et al. identified that the oncogenic RAB22A could be regulated by miR-193b in BC cells^79^. Exosomes lacking RAB22A diminish exosome-mediated growth, invasion, and migration of the recipient BC cells.

#### Exosomal proteins response to the hypoxic microenvironment

Hypoxic TME is a common characteristic of solid tumors and is often connected with aggressiveness and poor outcomes^80^. BC cells under hypoxic conditions secrete a large amount of exosomes, and that exosomes released from hypoxic BC cells promote focal adhesion formation, invasion, and metastasis, thus implicating that tumor-derived exosomes are mediators of tumor metastasis^81^. Sethuraman et al. proved that hypoxia-induced activation of BHLHE40 contributed to promoting cell survival and lung metastasis in vivo of BC by modulating exosomal secretion of heparin-binding epidermal growth factor (HBEGF)^82^. Therefore, the HIF-BHLHE40-HBEGF axis was an important signaling mechanism to promote metastasis of BC. Metastasis-associated protein 1 (MTA1) is a transcriptional co-regulator and is an upregulated protein in cancer, whose expression correlates with cancer development, unfavorable prognosis, and enhanced metastatic potential. Hannafon et al. identified the existence and confirmed the abundance of MTA1 in BC exosomes^83^. Functionally, MTA1 knockout in BC cells resulted in changes to hypoxia, and affected estrogen signaling and tamoxifen sensitivity that could be attenuated by the addition of MTA1 exosomes.

#### Exosomal proteins induce angiogenesis of tumor

BC-derived exosomes may transmit oncogenes and pro-angiogenic signals to stromal cells and thus promote stromal remodeling, tumor vascularization, and cell invasion^84^. Therefore, BC-exosomes render tumor cells more aggressive and more prone to metastasize. Annexin A2 (AnxA2), a 36-kDa calcium-dependent phospholipid-binding protein, was associated with various cancer-associated plasminogen activation, actin-cytoskeletal rearrangement, cellular adhesion, and migration^85^. The exosomal AnxA2 was significantly higher in malignant cells than in normal and pre-metastatic BC cells^86^. Moreover, in vivo analysis indicated that the exosomal AnxA2-depleted exosomes decreased the metastatic sites of the brain and lung, confirming the important role of exosomal AnxA2 in creating a favorable TME for metastasis. High expression of circulating exosomal AnxA2 was also associated with tumor grade and resulted in poor survival of the BC patients^87^. This suggested that the circulating exosomal AnxA2 could predict the prognosis of BC patients. In vivo Matrigel plug assay further demonstrated that a high level of exosomal AnxA2 in serum was potently induced angiogenesis in BC patients. Therefore, BC exosomal AnxA2 promoted angiogenesis and organ-specific metastasis of BC. In 2020, Liu et al. verified the specific highly active deubiquitinase ubiquitin carboxyl-terminal hydrolase isozyme L1 (UCHL1) as a candidate oncoprotein in the aggressive BC patient serum and BC-cell ﻿conditioned media (CM) by deubiquitinase activity profile^88^. Besides, UCHL1 was specifically enriched in donor cell exosomes to promote migration and extravasation in recipient cells by upregulating TGFβ/SMAD signaling pathways, which could be antagonized by UCHL1 inhibitor. This result highlighted the significance of UCHL1-containing exosomes serving as novel blood markers for early diagnosis of aggressive BC.

#### Exosomal proteins mediate disruption of vascular barrier

The endothelial barrier strictly maintains the homeostasis balance of blood vessels and tissues, thus regulating many processes, such as angiogenesis, immune response, and tumor invasion^89^. Although intravasation depends on the migration ability of tumor cells themselves, the disruption of vascular barrier integrity facilitated by molecular changes is also important to promote the ability of BC cells to cross the endothelial cell barriers. TNBC is characterized by high levels of stromal and intratumoral tumor-infiltrating lymphocytes (TILs), which is a potential predictive biomarker of more favorable survival outcomes and response to chemotherapy for immunotherapy response in TNBC. The crosstalk between lymphocytes and tumor cells was crucial for the modulation of the secretion in the exosomal mRNA profile of recipients. Theodoro et al. conducted an in vitro assay that the lymphocytes collected from healthy women were co-cultured with BC cells MCF-7 to stimulate heparinase expression^90^. The results revealed that BC-derived heparan sulfate (HS) could be carried by exosome particles, leading to upregulation of ﻿heparanase (HPSE) and HPSE2 expression of lymphocytes, which was highly relevant for cellular migration and tumor metastasis. Blomme et al. also showed that the cancer-overexpressed protein myoferlin was present in exosomes derived from BC cells to promote cell migration and invasion^91^. The depletion of myoferlin in cancer exosomes significantly modulated exosomal protein load, and reduced the capability to induce HUVEC migration and proliferation. Thrombospondin-1 (TSP1) is a multifunctional ECM glycoprotein, generally regulates the signaling pathways of CD47, CD36, and TGF-β to promote tumor progression in various malignancies^92^. Cen et al. showed that BC-derived exosomal TSP1 facilitated the trans-endothelial migration of BC cells by disrupting the intercellular integrity of endothelial cells, and by reducing the expression of intercellular junction proteins VE-cadherin and ZO-1 both in vitro and in vivo^93^.

#### Exosomal proteins mediate colonization of targeted organ

Nephronectin (NPNT) was the primary ligand for α8β1 integrin and was crucial in kidney development and had intricating roles in BC progression and metastasis. In 2018, Steigedal et al. showed that NPNT might be localized in mouse BC-cell-derived exosomes and display the ability to increase adhesion and anchorage-independent growth in vitro in an integrin-dependent manner^94^. The disruption of the integrin-binding site within NPNT could regulate the capability of mBC cells to adhere and colonize in the lung. This study presumed that there was a link between NPNT-containing exosomes and enhanced metastatic capacity in vivo. Zhang et al. demonstrated that POPX2 was found to be associated with BC-cell invasiveness and lung colonization in the early stages of metastasis^95^. Knockdown of POPX2 promoted exosome secretion to modulate the cytokine secretome including angiogenic proteins, such as fibroblast growth factor (FGF) and platelet-derived growth factor (PDGF), thus contributing to tumor angiogenesis at later stages of metastasis.

BCBM often induces neurological impairments by affecting cognitive and sensory functions and confer a poor prognosis, high mortality, and lack of effective therapy. A comprehensive understanding of tumor-intrinsic properties and drivers of the crosstalk that allows BC cells to infiltrate the brain is critical to prevent and treat BCBM. BC-derived exosomes were essential in remodeling the brain microenvironment during metastatic colonization. The cell migration-inducing protein (CEMIP), also called KIAA1199, had increased expression in cancers and functioned as a regulator related to cell survival, growth, and invasion^96^. CEMIP was screened in exosomes released by viable brain metastatic tissues from breast and lung cancer patients and its high expression in human primary and metastatic tumors was obviously related to the accelerated metastatic progression and poor survival rate^97^. CEMIP was abundant in exosomes from additional orthotopic brain metastatic models of BC. It was interesting that tumor-derived exosomal CEMIP not only remodeled brain vasculature but also induced inflammatory brain vascular niches to support brain metastasis. Furthermore, the exosomal CEMIP pretreatment promoted brain metastatic colonization, regaining the ability of CEMIP-depleted cells to associate with brain vasculature.

Significant changes in protein expression have been observed during the differentiation and maturation of osteoclasts, which can be used as an emerging circulating biomarker for bone metastasis. The pathogenesis of bone metastasis depends on cross-communication between tumor cells and various stromal cells such as osteoblasts, osteoclasts, and mineralized bone matrix. The cytosolic protein L-plastin could be released in BC-derived exosomes^98^. The BC exosomal L-plastin by BC cells mediated osteoclast activation and facilitated metastatic bone osteolysis, inferring the critical role of L-plastin in stimulating osteoclastogenesis and promoting osteolysis during BC bone metastasis. Integrin β3 (ITGB3) belongs to a family of transmembrane proteins that integrate the ECM processes and participates in reprogramming tumor metabolism. ITGB3 knockdown led to a decreased cellular energy metabolism, suppressed tumor growth, impaired cytokinesis, and migration possibilities, and decreased vesicle trafficking^99^. It was speculated that the level of ITGB3 was upregulated in exosomes derived from the MDA-MB-231 in the skeletal metastasis model, and functioned as a crucial molecule in the formation of pre-metastatic niche adhesion by mediating local and distal effects via direct contact or exosomes.

### Exosomal metabolites

Reprogrammed energy metabolism is a hallmark for tumor features. Energetic requirements and excretions exhibit huge unbalance in tumor cells to mediate tumor growth and metastasis through metabolite transfer and metabolic coupling. Emerging studies have demonstrated that the proteome and transcriptome of tumor metastasis are dynamically regulated by the metabolome. Metabolome-induced signaling cascades may drive tumor aggression and metastasis via diverse signal-regulating roles of metabolites in the complex process of metastasis^100^. It proposed that ketone body production and re-utilization could drive more enhanced BC growth and metastasis, as Martinez-Outschoorn et al. reported that ketogenic fibroblasts could modify MDA-MB-231 cells that overexpressed the enzyme required for ketone re-utilization to be a more invasive phenotype^101^. Similarly, Ko et al. showed that energy-rich glutamine was an essential molecular from autophagic fibroblasts to favor BC-cell mitochondrial activity, and encouraged a pernicious cycle of catabolism in the tumor stroma and anabolic tumor cell dissemination^102^. Exosomes are efficient carriers to encapsulate metabolites, including lipids, lactate, glutamate, acetate, stearate, palmitate, and amino acids^103^. For example, Puhka et al. implemented metabolomic analysis in the urinary exosome-containing extracellular vesicles from the prostate patients, indicating that the levels of glucuronate, D-ribose 5-phosphate, and isobutyryl-L-carnitine were significantly lower in prostatectomy samples than healthy control^104^. This study was potential evidence that the exosomal cargoes were far more complicated and contained various types of metabolites. Exosome-derived metabolites might respond to the extracellular environment and might be transporters to fuel rapid cell growth and proliferation.

Lipids are involved in exosome biosynthesis and are key components of exosomal membranes. Specifically, exosomes are rich in lipids such as sphingomyelin, ceramide, phosphatidylserine, cholesterol, and saturated fatty acids, and most of them are located on membrane^105^. Compared with their parental cells, specific lipids are located in exosomes and therefore have the potential to serve as identification markers^106^. Docosahexaenoic acid (DHA) is a long-chain, highly unsaturated omega 3 fatty acid with anti-angiogenesis effects. In vitro assay, exogenous DHA treatment downregulated the expression level of pro-angiogenic genes and miRNA (upregulated miR-101, miR-199, miR-342, and downregulated miR-382, miR-21) in MDA-MB-231 cell and their exosomes^107^. Some studies have verified that different mechanisms of exosomal lipids and the variability are involved in cancer diseases. Fatty acid encapsulated in tumor-derived exosomes could promote immune evasion by inducing dysfunctional dendritic cells (DC)^108^. The cholesterol metabolite 27-Hydroxycholesterol (27-OHC) was involved in proliferation and metastasis in ﻿estrogen receptor-positive (ER+) BC via the ER receptor and liver X receptor (LXR), respectively^109^. Compared with exosomes derived from estrogen receptor-negative (ER−) BC-cell line (MDA-MB-231) and other control, the exosomal 27-OHC from ER+ BC-cell line (MCF-7) were significantly increased^110^. Thus, lowering circulating cholesterol levels like 27-OHC may be a useful strategy to prevent and/or treat BC. However, the value of exosomal 27-OHC in BC diagnosis was still lacking direct confirmation.

To sum up, these molecular metabolites are also encapsulated and shuttled to adjacent cells via BC-derived exosomes. At present, most researches focus on exosomes containing certain proteins and RNAs. However, although many metabolites participate in BC progression, it is a pity that there are few studies about the metabolites carried by tumor-derived exosomes in BC. Generally speaking, the studies on the role of exosomal nucleic acids and proteins relatively are much more than metabolites in the tumor progression, partly because nucleic acids and proteins possess more genetic specificity to regulate recipient cell behaviors via exosome-mediated transportation. More studies are needed to decipher the role of exosomal lipids and amino acid metabolism to regulate tumor progression.

## BC-derived exosomes in BC metastasis immune regulation

BC cells generate an immunosuppressed TME, which is a very highly intricate and heterogeneous system, incorporating cancer cells, CAFs, endothelial cells, immune cells, ECM, and various cytokines. TME is associated with induction of proliferation, angiogenesis, apoptosis inhibition and immune system suppression, and drug resistance^111^. Especially, induction of antitumor immunity to resist immune suppression is one of the most breathtaking strategies for therapeutic use for tumor therapy. Escaping immune surveillance is a hallmark and prerequisite for the establishment of a permissive environment in the secondary organs of the primary tumor, thereby enabling metastasis and sustaining tumor progression. In the immune system, exosomes are fundamental mediators secreted by each immune cell type to fulfill its function and promote inflammation or tolerance. The activities of exosomes affect multiple immunological behaviors, including modulating antigen presentation, immune activation and suppression, immune surveillance, and intercellular communication^112^. It is well known that tumor-derived exosomes can exert detrimental effects on the immune system by impairing specific T-cell immunity and bias innate immune cells toward a tumor-promoting phenotype. Clearly, tumor-derived exosomes impact recipient immune cells with the complex nature of those interactions and the molecular drivers to propel tumor metastasis. The inhibition of tumor-derived exosome uptake or carried components is of value to restore function in immune-suppressed cells.

BC exosomes travel to specific homing niches dynamically modify gene expression and molecular architectures to mediate immune lymphocyte migration, host defense, and tumor metastasis^113^. Hoshino et al. verified that BC exosomes expressing high levels of *αv*β5 were distributed to liver tissue containing FN-enriched ECM, whereas those with high levels of integrin α6β4 were distributed to the lung tissue containing ECM enriched with laminin^114^. The exogenously fluorescently labeled exosomes of highly-metastatic murine BC cells were recruited predominantly to mice lung where BC metastases were frequent, speculating BC exosome accumulation made the lung environment more prone to metastatic tumor colonization^115^. Moreover, the BC-derived exosome showed a stimulative effect on myeloid-derived suppressor cells (MDSC) accumulation and a direct suppressive effect on T-cell proliferation and NK cell cytotoxicity, and hence likely weakened the antitumor immune response in pre-metastatic niches. Therefore, the tissue-specificity of BC-derived exosome accumulation contributed to immune suppression and increased metastatic colonization. Jiang et al. proposed that BC exosomal miR-9 and miR-181a were upregulated in early-stage MDSCs to promote the early-stage MDSCs amplification and T-cell immunity suppression, leading to promote tumor growth^116^. This process was mediated via JAK/STAT signaling pathway activation by targeting SOCS3 and PIAS3, respectively. Deng et al. found that tumor cell interaction with tumor-associated leukocytes was required for induction of tumor-exosomal FN^77^. The tumor-derived exosomal FN promoted 4T1 breast tumor invasion and metastasis in vivo, which could be regulated by CD25+ cells and Gr-1+ cells by recruiting FN into exosomes.

Since tumor-derived exosomes could target immune cells to induce an immunosuppressive tissue environment, providing the potential of serving as targets for novel anticancer therapies for BC. The engineered BC exosome modified with overexpressed miRNAs had potential anticancer effects as cell-free vaccine for BC treatment^117^. The BC exosome manipulated Let-7i, miR-142, and miR-155 synergistically and efficiently induced the DC maturation and promote tumor escape^117^. BC exosomes carried programmed death-ligand 1 (PD-L1) were highly immunosuppressive in BC TME. TGF-β acted as a promoter of exosomal PD-L1 to promote CD8+ T-cell dysfunction. This result proclaimed that TGF-β espoused tumor-derived exosomal PD-L1 to attenuate phosphorylation of Src family proteins of activated CD8+ T cells and promote CD8+ T-cell dysfunction^118^. These results all confirmed the significance for the roles of tumor-derived exosomes in communicating with immune cells, involved in DC differentiation and maturation, CD8+ T-cell dysfunction, and regulatory immune cells like MDSCs and Tregs. More comprehensive proteomic and RNA profiling studies of BC-derived exosomes could help identify key exosome contents that drive immune regulation.

## Conclusions and expectations

**Fig. 3:**
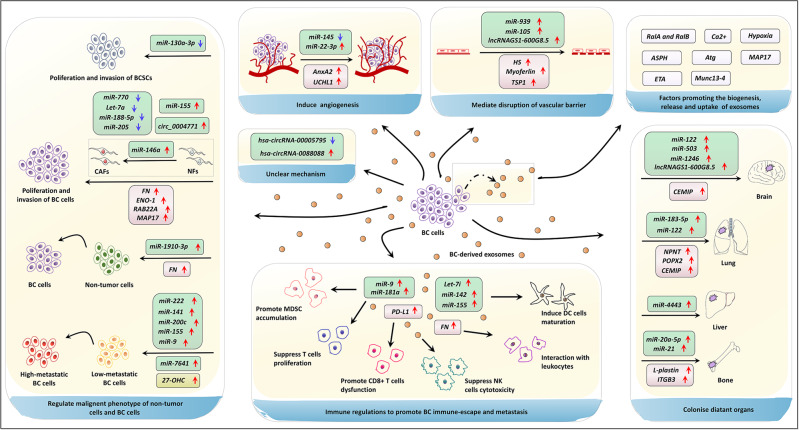
The multifaceted roles and mechanisms of tumor-derived exosomal components in breast cancer metastasis. Exosomes could mediate metastatic initiation and progression of BC by transferring functional cargos. BC-derived exosomes can modify the malignant phenotype of non-tumor cells and low-metastatic BC cells such as promoting proliferation and invasion of BCSCs, promoting proliferation and invasion of BC cells through the transformation of NFs to CAFs. Besides, the specific cargos are also involved in metastasis via inducing angiogenesis and disruption of vascular permeability. Furthermore, tumor-derived exosomes participate in organotypic metastasis. Through lymphatic circulation and blood circulation, primary BC-derived exosomes can transfer proteins (e.g., NPNT) and nucleic acids (e.g., miRNAs) to distant organs, thus inducing colonization and infiltration of new sites. The key procedure for establishing a permissive environment for BC metastasis is also related to interaction between BC cells and immune cells to escape immune surveillance. For example, BC exosomes carried PD-L1 could promote CD8+ T-cell dysfunction to facilitate immune-suppressing and metastasis. Most studies have confirmed the expression levels and metastasis capabilities of cargos in exosomes involving in BC metastasis, while a few studies only confimed the genetic changes of cargoes by sequencing. Finally, the role of exosomes in BC metastasis is also associated with factors affecting exosomal biogenesis, release and uptake. For example, hypoxia and ETA can promote BC metastasis by promoting the release of exosomes. ↑ upregulation, ↓ downregulation, breast cancer BC, human breast cancer stem cells BCSCs, normal fibroblasts NFs, cancer-associated fibroblasts CAFs, interleukin IL, tumor necrosis factor TNF, microRNA miRNA, long noncoding RNA lncRNA, circular RNA circRNA, extracellular matrix protein nephronectin NPNT, cell migration-inducing and hyaluronan-binding protein CEMIP, ubiquitin carboxyl-terminal hydrolase isozyme L1 UCHL1, integrin β3 ITGB3, 27-hydroxycholesterol 27-OHC, thrombospondin-1 TSP1, fibroblast growth factor FGF, platelet-derived growth factor PDGF, annexin A2 AnxA2, enolase-1 ENO-1, heparan sulfate HS, autophagy and autophagy-related genes Atg, endothelin receptor A ETA, asparaginyl β-hydroxylase ASPH, myeloid-derived suppressor cells MDSC, dendritic cells DC, and programmed death-ligand 1 PD-L1.

Exosomes have gained extensive attention in cancer due to the multifaceted roles such as remodeling TME, reshaping tumor progression, and conferring therapy resistance. The current studies have partially elucidated the behaviors and mechanisms of BC-derived exosomes in reprogramming BC metastasis, which principally ascribed to the content of tumor exosome and the bioactive cargoes encapsulated in exosomes. In this review, we have found that the exosomal miRNA (miR-146a, miR-1910-3p, miR-9, and miR-155, etc.), lncRNA (lncRNAGS1-600G8.5, hsa-circRNA-0088088, etc.), circRNA, protein (MTA1, UCHL1, AnxA2, TSP1, etc.), lipid (27-OHC), were the main active components being studied in BC metastasis, as listed in Table 1. The organotypic metastasis induced by these BC exosomal components is achieved by regulating pre-metastatic niche formation, EMT, vascular permeability, angiogenesis, and colonization (Fig. 3).

**Table 1:** Outline of BC-derived exosomal cargos and biological functions, clinical values in BC metastasis.

Exosomal contents	Expression	Recipient cells	Mechanisms	Biological function	Clinic values	Ref.
*miR-130a-3p*	Downregulation	High-metastatic BC cells	Directly regulate RAB5B/EGFR signaling pathways	Inhibit BCSCs proliferation and migration	A noninvasive molecular marker for the diagnosis and prognosis of BC	^40^
*miR-770*	Downregulation	TAMs	Increase the STMN1 expression	Inhibit TNBC migration and invasion	A prognostic biomarker in TNBC	^41^
*Let-7a*	Downregulation	High-metastatic BC cells	Silence the c-Myc expression	Inhibit the proliferation and migration of TNBC cells	A possible therapy target of BC	^42^
*miR-188-5p*	Downregulation	High-metastatic BC cells	Selective sorting into exosomes and regulate its target IL6ST	Inhibit BC-cell migration	A possible diagnostic biomarker of BC	^43^
*miR-155*	Upregulation	Adipocytes and muscle cells	Induce lipolysis and muscle loss	Leading to cancer-associated cachexia	A possible therapy target of BC	^44^
*miR-222*	Upregulation	Low-metastatic BC cells	Downregulate tumor suppressor gene PDLIM2 and activate NF-kB	Correlate to BC patients with lymphatic metastasis	A potential approach of therapeutic strategies for advanced BC	^45^
*miR-146a*	Upregulation	NFs	Enhance the transformation of NFs into CAFs via TXNIP/Wnt pathways	Accelerate invasion of BC cells	A possible therapy target of BC	^46^
*miR-1910-3p*	Upregulation	Mammary epithelial cells	Regulate MTMR3 and activate the NF-κB signaling pathway	Promote growth and metastasis of BC cells	A new molecular marker for BC diagnosis	^47^
*miR-200c miR-141*	Upregulation	N/A	N/A	N/A	Potential biomarkers for early detection of BC metastasis	^48^
*miR-205*	Downregulation	N/A	Potentiate the inflammatory responses	Attenuate BC metastasis	A possible therapy target of BC	^50^
*miR-9*	Upregulation	Low-metastatic BC cells	Target tumor suppressors PTEN	Enhance metastatic phenotype of BC	A possible therapy target of BC	^51,52^
*miR-155*	Upregulation	Low-metastatic BC cells	Potentiate the inflammatory responses; Target tumor suppressors DUSP14	Enhance metastatic phenotype of BC	A possible therapy target of BC	^50–52^
*miR-7641*	Upregulation	Low-metastatic BC cells	Induce epigenetic modulation	Promote BC-cell proliferation and invasion	A diagnostic biomarker and therapy target of BC	^53^
*miR-22-3p*	Upregulation	HUVECs	Mediate tumor vessel abnormalization by suppressing transgelin	Promote tumor budding and BC progression	A potential therapeutic target for BC	^54^
*miR-145*	Downregulation	HUVECs	Target IRS1 and inhibit the angiogenesis of HUVECs	Weaken angiogenesis	A possible therapy target of BC	^21^
*miR-939*	Upregulation	Endothelial cells	Target VE-cadherin	Increase tumor cell trans-endothelial migration	A possible therapy target of BC	^55^
*miR-105*	Upregulation	Endothelial cells	Damage the integrity of natural barriers by targeting ZO-1	Benefit for BC metastasis	Biomarkers in the circulation	^56^
*miR-21 miR-105*	Upregulation	N/A	N/A	N/A	Potential biomarkers for BC metastasis	^57^
*miR-20a-5p*	Upregulation	BMMs	Facilitate the osteoclastogenesis by targeting SRCIN1	Promote the migration and invasion of MDA-MB-231 cells	﻿A potential target for BC therapy	^58^
*miR-21*	Upregulation	Osteoclast	Boost osteoclastogenesis via regulating PDCD4 protein levels	Promote BC bone metastasis	A potential target for BC therapy and diagnosis	^59^
*miR-183-5p*	Upregulation	Macrophages	Promote the secretion of pro-inflammatory cytokines IL-1β, IL-6, and TNF-α via repressing PPP2CA	Promote BC growth and metastasis	A potential target in tumor therapies	^60^
*miR-33*	Downregulation	Macrophages	Induce M1 polarization	Inhibit BC growth and metastasis	A potential target for BC therapy	^61^
*miR-4443*	Upregulation	﻿Stromal cells	Break the natural barriers via impairing TIMP2 and consequently upregulating MMP-2	Induce BC liver metastasis	A new therapy to inhibit liver metastasis of BC	^38^
*miR-122*	Upregulation	Non-tumor cells	Suppress the glucose uptake of non-tumor cells	Reprogram glucose consumption and promote metastasis	A predictive marker and a therapeutic target for metastatic BC	^62^
*miR-503*	Upregulation	Microglia	Promote M1–M2 polarization by modulating the STAT 3 and NF-kB pathways	Promote BC brain metastasis	A promising biomarker of BC	^63^
*miR-1246*	Upregulation	Non-tumor cells	Inhibit the expression level of cyclin-G2	Boost cell proliferation and invasion of BC	A serum biomarker for BC	^64,65^
*lncRNA BCRT1*	Upregulation	Macrophages	Promote M2 polarization	Promote BC metastasis	A potential therapeutic target and prognostic predictor	^70^
*lncRNAGS1-600G8.5*	Upregulation	Brain microvascular endothelial cells	Disrupt the permeability of BBB by inhibiting tight junction proteins	Induce BC brain metastasis	A promising therapeutic target for BC brain metastasis	^71^
*hsa-circRNA-00005795*	Downregulation	N/A	N/A	Involve in BC metastasis	A new biomarker for predicting metastasis of BC	^73^
*hsa-circRNA-0088088*	Upregulation	N/A	N/A	Involve in BC metastasis	A new biomarker for predicting metastasis of BC	^73^
*circ_0004771*	Upregulation	N/A	harbor miR-1253 in BC to upregulate DDAH1	Alter BC malignant phenotypes	A potential target for BC therapy	^74^
*Cd38* *Slco2a1* *Acsl4* *Mtdh* *Fgfr* *Tgfbr*	Upregulation	N/A	N/A	Involve in high BC metastasis	Potential biomarkers for BC and as targets to block metastasis	^75^
*FN*	Upregulation	N/A	Interact with tumor-associated leukocytes	Promote 4T1 breast tumor metastasis	Novel therapeutic strategies	^77^
*ENO-1*	Upregulation	N/A	gather proteolytic activity on the BC-cell surface	Promote BC progression	N/A	^78^
*MAP17*	Upregulation	Low-metastatic BC cells	Increase the EMT	Promote BC metastasis	A novel target for therapy of mBC	^32^
*HBEGF*	Upregulation	Low-metastatic BC cells	Hypoxia induce activation of BHLHE40, and promote exosomal secretion of HBEGF	promote BC lung metastasis	Therapeutic target for BC	^82^
*MTA1*	Upregulation	Low-metastatic BC cells	Changes to hypoxia and affect estrogen signaling	Enhance BC metastasis	Potential therapeutic strategies for BC	^83^
*AnxA2*	Upregulation	Stromal cells; Endothelial cells	Create favorable TME via activation of p38, NF-ĸB, and STAT 3 pathways; promote angiogenesis	Promote BC brain and lung metastasis	Predict prognosis of BC patients	^86,87^
*UCHL1*	Upregulation	Low-metastatic BC cells	Upregulate TGF-β/SMAD signaling pathways and promote EMT	Promote BC migration and extravasation	A novel blood marker for early diagnosis of BC	^88^
*HS*	Upregulation	N/A	Upregulation of HPSE and HPSE2 expression in lymphocytes	Promote BC metastasis via crosstalk with circulating lymphocytes	A target for tumor prognosis and treatment for BC	^90^
*Myoferlin*	Upregulation	Endothelial cells	Alter protein content	Induce HUVEC migration and proliferation	N/A	^91^
*TSP1*	Upregulation	Endothelial cells	Disrupt the intercellular integrity; reduce the expression of VE-cadherin and ZO-1	Facilitate the trans-endothelial migration of BC cells	N/A	^92^
*NPNT*	Upregulation	Non-tumor cells	Increase adhesion and anchorage-independent growth in an integrin-dependent manner	Promote metastatic BC cells to adhere and colonize in the lung	A potential prognostic marker and a target for BC therapy	^94^
*FGF* *PDGF*	Upregulation	Endothelial cells	Promote angiogenesis	Promote BC metastasis	N/A	^95^
*CEMIP/ KIAA1199*	Upregulation	Low-metastatic BC cells, brain stromal cells	Remodel brain vasculature and induce inflammatory brain vascular niche	Support BC brain metastasis	A promising prognostic biomarker and therapeutic target for mBC	^96^
*L-plastin*	Upregulation	Non-tumor cells	Stimulate osteoclastogenesis and promote osteolysis	Activate osteoclast and facilitate metastatic bone osteolysis	A diagnostic and prognostic marker	^98^
*ITGB3*	Upregulation	Low-metastatic BC cells	Modulate expression of multiple gene about cancer metastasis	Promote BC metastasis	A target for treating BC skeletal metastasis	^99^
*27-OHC*	Upregulation	ER-BC cells	N/A	Promote BC metastasis	A useful strategy to treat BC	^110^
*miR-9 miR-181a*	Upregulation	Early-stage MDSCs	Mediate JAK/STAT signaling pathway activation by targeting SOCS3 and PIAS3	Promote BC growth and metastasis	A possible therapy target of BC	^116^
Let-7i miR-142 miR-155	Upregulation	DC	induce the DC maturation	promote BC immune-escape	A possible therapy target of BC	^117^
PD-L1	Upregulation	CD8+ T-cell	Promote CD8+ T-cell dysfunction	promote BC metastasis	A possible diagnostic biomarker of BC	^118^

*N/A* Not applicable, *BC* breast cancer, *BCSCs* human breast cancer stem cells, *TAMs* tumor-associated macrophages, *TNBC* triple-negative breast cancer, *IL6ST* interleukin 6 signal transducer, *NFs* normal fibroblasts, *CAFs* cancer-associated fibroblasts, *TXNIP* thioredoxin-interacting protein, *MTMR3* myotubularin-related protein 3, *PTEN* phosphatase and tension homolog, *DUSP14* dual-specificity phosphatases 14, *VE-cadherin* vascular endothelial cadherin, *ZO-1* zona occluden-1, *PDLIM2* PDZ and LIM domain-containing protein 2, *SRCIN1* SRC kinase signaling inhibitor 1, *PDCD4* programmed cell death 4, *IL-1β* interleukin-1β, *TNF-α* tumor necrosis factor-α, *PPP2CA* phosphatase 2 catalytic subunit alpha, *TIMP2* tissue inhibitor of metalloproteinase 2, *MMPs* matrix metalloproteinases, *BBB* blood–brain barrier, *DDAH1* dimethylarginine dimethylaminohydrolase 1, *FN* fibronectin, *ENO-1* enolase-1, *EMT* epithelial–mesenchymal transition, *mBC* metastatic breast cancer, *HBEGF* heparin-binding epidermal growth factor, *MTA1* metastasis-associated protein 1, *AnxA2* annexin A2, *TME* tumor microenvironment, *UCHL1* ubiquitin carboxyl-terminal hydrolase isozyme L1, *HS* heparan sulfate, *HPSE* heparinase, *HUVEC* human umbilical vein endothelial cells, *TSP1* thrombospondin-1, *NPNT* nephronectin, *PDGF* platelet-derived growth factor, *CEMIP* cell migration-inducing protein, *ITGB3* integrin β3, *27-OHC* 27-hydroxycholesterol, *ER−* estrogen receptor-negative, *MDSC* myeloid-derived suppressor cells, *DC* dendritic cells, *PD-L1* programmed death-ligand 1.

However, there still are some challenges deserving of our attention in this area. Firstly, the BC metastasis ability is closely related to the molecular classification of BC. For example, TNBC and basal-like BC are of highly tumor metastasis characteristics and the prognosis is better for ER+ BC. In existing studies, the important relationship between exosomal cargoes and metastasis of specific BC subtype were not clinically emphasized and deserved further investigation. Secondly, TME is a complicated entity composed of multiple cell types and cell-secreted factors. That means, exosomes originated from different cell types orchestrate the tumor cell metastasis and the metastasis is the ending caused by these exosomal components and others synergistically. Therefore, it is difficult to judge the exosomes from which cell types occupy the leading position for BC metastasis. Thirdly, exosomal ncRNAs are evolving as important mediators in the interplay between the BC cells and TME. Although there are relatively many studies on miRNA, the studies on lncRNA and circRNA are still relatively insufficient. Besides, some ncRNA studies only explored the expression level by sequencing and PCR verification on tissue samples or cell lines, but lacking further investigation on metastasis mechanism. Fourthly, tumor-derived exosomes carry a wide range of nucleic acid and proteins, lipids, that can reflect the molecular contents of the parental cells. However, the abundance of these substances in exosomes is different from the expression level of parent cells, indicating that these substances are selectively encapsulated in exosomes in different cell types, including BC cells. And this phenomenon of selective encapsulation mechanism needs to be further addressed. Lastly, successful colonization of mBC cells requires reciprocal communications with stroma cells, endothelial cells, and local immune cells. The role and possible alteration of immune regulation in establishing pre-metastatic niches in future metastatic organs should be investigated.

For future perspectives, it will be meaningful to take consideration of tumor-derived exosomal biomarkers for BC diagnosis and therapy. Firstly, exosomes are promising candidates as biomarkers for early detection of BC and their prognosis. The conventional screening methods for BC including mammography, ultrasonography, PET-CT, and MRI, are usually costly and time consuming. The liquid biopsy is a classical and quick method by detecting various body fluids such as plasma, serum, urea, saliva, and tears. To some extent, exosomes loaded with specific bioactive molecules with differential expression in the above body fluids can represent the BC course and metastasis, thus posing the potential with sensitivity and specificity to detect early metastasis and predict prognosis for BC patients by liquid biopsy. Secondly, undoubtedly, tumor-derived exosomes are potential therapeutic targets in BC therapy, including metastasis intervention, drug resistance, and drug delivery. Blocking the release or the key components of exosomes associated with metastasis represents a new approach for developing anti-BC therapeutic agents, which might be used alone or in conjunction with traditional therapies.

Further works are needed to illuminate the potential mechanisms of exosomal components in BC metastasis. The profound understanding of tumor-exosomal components in reshaping the properties of BC metastasis, will undoubtedly provide novel insights for developing diagnosis and therapy strategies.

## Data Availability

Not applicable.
